# The Choroid Plexus Is Permissive for a Preactivated Antigen-Experienced Memory B-Cell Subset in Multiple Sclerosis

**DOI:** 10.3389/fimmu.2020.618544

**Published:** 2021-01-26

**Authors:** Jürgen Haas, Henriette Rudolph, Leonardo Costa, Simon Faller, Saskia Libicher, Cornelia Würthwein, Sven Jarius, Hiroshi Ishikawa, Carolin Stump-Guthier, Tobias Tenenbaum, Christian Schwerk, Horst Schroten, Brigitte Wildemann

**Affiliations:** ^1^ Molecular Neuroimmunology Group, Department of Neurology, University Hospital of Heidelberg, Heidelberg, Germany; ^2^ Department of Pediatrics, Pediatric Infectious Diseases, Medical Faculty Mannheim, Heidelberg University, Heidelberg, Germany; ^3^ Laboratory of Clinical Regenerative Medicine, Department of Neurosurgery, Faculty of Medicine, University of Tsukuba, Tsukuba, Japan

**Keywords:** multiple sclerosis, blood-cerebrospinal fluid barrier, B lymphocytes, transmigration, human

## Abstract

The role of B cells in multiple sclerosis (MS) is increasingly recognized. B cells undergo compartmentalized redistribution in blood and cerebrospinal fluid (CSF) during active MS, whereby memory B cells accumulate in the CSF. While B-cell trafficking across the blood–brain barrier has been intensely investigated, cellular diapedesis through the blood–CSF barrier (BCSFB) is incompletely understood. To investigate how B cells interact with the choroid plexus to transmigrate into the CSF we isolated circulating B cells from healthy donors (HC) and MS patients, utilized an inverted cell culture filter system of human choroid plexus papilloma (HIBCPP) cells to determine transmigration rates of B-cell subsets, immunofluorescence, and electron microscopy to analyze migration routes, and qRT-PCR to determine cytokines/chemokines mediating B-cell diapedesis. We also screened the transcriptome of intrathecal B cells from MS patients. We found, that spontaneous transmigration of HC- and MS-derived B cells was scant, yet increased significantly in response to B-cell specific chemokines CXCL-12/CXCL-13, was further boosted upon pre-activation and occurred *via* paracellular and transcellular pathways. Migrating cells exhibited upregulation of several genes involved in B-cell activation/migration and enhanced expression of chemokine receptors CXCR4/CXCR5, and were predominantly of isotype class switched memory phenotype. This antigen-experienced migratory subset displayed more pronounced chemotactic activities in MS than in HC and was retrieved in intrathecal B cells from patients with active MS. Trafficking of class-switched memory B cells was downscaled in a small cohort of natalizumab-exposed MS patients and the proportions of these phenotypes were reduced in peripheral blood yet were enriched intrathecally in patients who experienced recurrence of disease activity after withdrawal of natalizumab. Our findings highlight the relevance of the BCSFB as important gate for the entry of potentially harmful activated B cells into the CSF.

## Introduction

The central nervous system (CNS) is separated from blood-derived leukocytes and soluble factors by complex barrier systems, among them the blood–brain barrier (BBB), which encompasses deep parenchymal microvessels; the blood–meningeal barrier (BMB); and the blood–cerebrospinal fluid (CSF) barrier (BCSFB), which encases the choroid plexus, epithelium-based structures localized within the brain ventricles that produce CSF (1–4). The BBB is composed of tight-junction-interconnected endothelial cells that, on their abluminal side, are ensheathed by the glia limitans perivascularis, an astroglial endfeet structure. Whereas the BBB requires an inflammatory environment to permit extravasation and transmigration of immune cells (1–4) the BCSFB, which comprises tight junction-conjoined cuboidal epithelial cells surrounding a core of stroma vascularized with fenestrated capillaries, enables low-level homeostatic cell trafficking even under physiological conditions and thus allows continuous leukocyte surveillance in the CSF compartment (3, 4). Notably, it has been postulated that the BCSFB functions as the primary gate governing the entry and subsequent parenchymal invasion of autoreactive immune cells in experimental autoimmune encephalomyelitis (EAE), the animal model of multiple sclerosis (MS) (5). As a consequence, understanding how the BCSFB functions under normal and inflammatory conditions is essential to elucidate the initial mechanisms driving MS pathogenesis, where heightened influx of immune cells prompts autoimmune damage to CNS tissues (6, 7). In humans, the multistep diapedesis process that governs leukocyte trafficking across the BCSFB is less well understood than the migration of immune cells into the brain parenchyma through the BBB, and has, so far, been studied exclusively in T cells (8, 9). However, accumulating evidence favors a pathogenic role of B cells and antibodies in MS. This is convincingly emphasized by the remarkable therapeutic effect of B-cell-depleting antibodies on clinical and radiological disease activity (10–12), and other key features such as persistent intrathecal production of immunoglobulins in the CSF, histological findings demonstrating immunoglobulin and complement deposits in a subset of inflammatory brain lesions, and the presence of follicle-like structures in the meninges detectable in some patients with progressive MS, as well as traceable autoantibodies against neural antigens in subsets of patients (13–16). While barely detectable in normal CSF, B-lineage cells are clonally expanded in CSF and brain tissues from MS patients (17–20). Indeed, this process appears to be driven by a compartmentalized redirection of memory B-lineage cells from blood into CSF, which paves the way for local conversion into antibody-secreting effector cells, as we have shown in a previous study (21). These findings strongly emphasize a role of the BCSFB in initiating and perpetuating aberrant CNS B-cell-mediated immune responses in MS. Here, we used an established *in vitro* model of the choroid plexus to identify the determinants of human B-cell trafficking across the BCSFB both under physiological conditions and in the context of MS (22–25). We also aimed to decipher the key B-cell subset involved in shifting between the systemic and intrathecal compartments.

## Methods

### Study Design

The aim of this study was to investigate migration of human B lymphocytes through the choroid plexus. An inverted transwell culture system of human choroid plexus papilloma (HIBCPP) cells was used as an *in vitro* model of the choroid plexus to determine transmigration rates of B-cell subsets. Immunofluorescence and electron microscopy were performed to analyze diapedesis routes through HIBCPP cells. We conducted further experiments to screen the transcriptome of *in vitro* migrated B cells as well as of intrathecal B cells obtained from MS patients by using a PCR array technique. Blood and CSF sampling was approved by the local ethics committee and all subjects signed informed consent. Group sizes were selected on the basis of our experience with these systems. All human samples were de-identified. Investigators were not blinded when conducting or evaluating the experiments and no randomization was necessary. No data were excluded from this study.

### Human Samples

The study included 60 healthy control donors (HC, mean age 34.2 years, range 18–60 years) and 30 patients with the relapsing-remitting form of MS (RRMS) according to the revised McDonald criteria (26). All MS patients were recruited at the outpatient clinic of the Department of Neurology, University of Heidelberg, Germany. 20 patients received no treatment for at least 2 months before recruitment, while ten patients were exposed to fingolimod (n = 5); 6, 6, 10, 15, and 18 months of treatment) or natalizumab (n = 5); 15, 22, 34, 46, and 71 months of treatment) at the time of blood sampling. Patients had a mean age of 34.1 years (range 22–55), a disease duration of 5.0 years (1–22), an Expanded Disability Status Scale (EDSS) score of 2.5 (0–4) and on average 2.5 (0–12) previous relapses. Twelve patients had clinically active disease, and 18 patients were in clinical remission (untreated n = 8, treated n = 10). Dysfunction of the BCSFB as depicted by the CSF to serum ratio of albumin (Q_Alb_) was present in one case only. We further included flow cytometric data from parallel blood and CSF samples of four RRMS patients before and following cessation of natalizumab selected from an independent cohort [mean age 38.4 years, range 35–45 years; disease duration: 7.2 years (3–24); EDSS: 3.0 (2–4); previous relapses: 3.5 (2–7); treatment duration: 18, 27, 42, and 50 months]. The study design was approved by the ethics committee of the University Hospital Heidelberg. Written informed consent was obtained from all study participants.

### Sampling/Cell Separation

From all study participants peripheral blood mononuclear cells (PBMCs) were isolated from 10 to 50 ml peripheral blood by Ficoll-gradient centrifugation (Biochrom, Berlin, Germany). Cerebrospinal fluid (CSF) samples (0.5–4.5 ml) were obtained from a subcohort of eight study patients (all in acute relapse and treatment-naïve). Total B cells were purified from PBMCs and from CSF cells with CD19 Microbeads (Miltenyi Biotec, Bergisch-Gladbach, Germany) or Dynabeads Untouched Human B cells Kit (Thermo Fisher, Dreieich, Germany).

### Transmigration Assay

**Figure 1:**
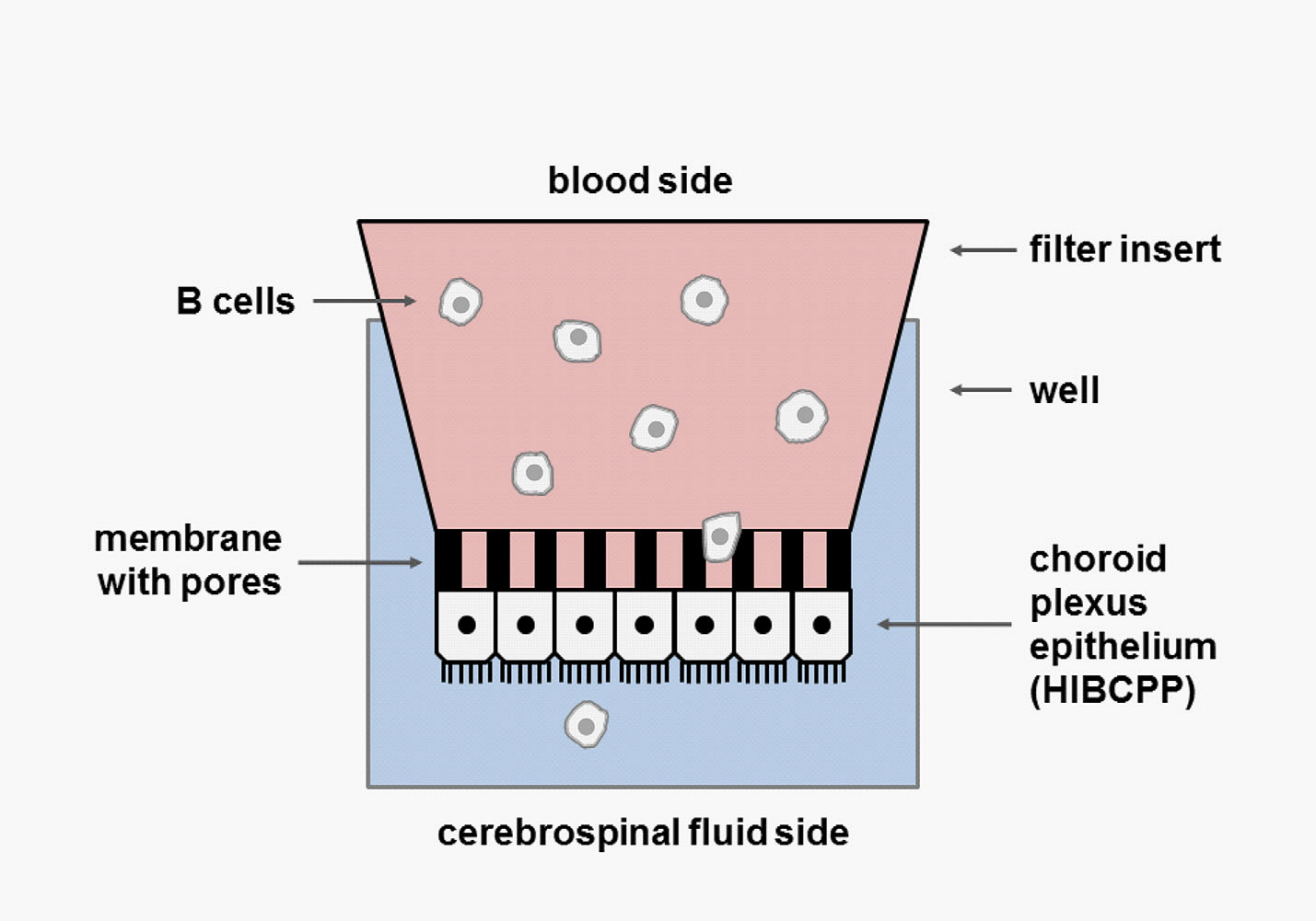
Inverted transwell culture system. Inverted culture of HIBCPP cells on the bottom side of a transwell filter of 5 µm pore size. The upper (basolateral) side represents the lumen of the blood capillary, while the lower (apical) side mimics the CSF space. B cells are added to the upper compartment (blood side) and a cocktail of B-cell chemoattractants to the lower compartment. After 4 h transmigration (TM), filters are transferred onto a fresh plate; migrated cells are collected from the lower chamber and quantified by FACS. A chemotactic index (CI) is calculated for each B-cell subset.

B-cell transmigration (TM) across the BCSFB epithelium was assessed using an established inverted transwell culture system (**Figure 1**) (22–24). Briefly, 5 × 10^4^ human choroid plexus papilloma (HIBCPP) cells (27), cultured in DMEM/F12 supplemented with 10% fetal calf serum (FCS), 0.5 μg/ml insulin, 100 U/ml penicillin, and 100 μg/ml streptomycin, were seeded on the bottom side of a 5-µM pore size transwell filter (Millipore, Schwalbach, Germany). TM filters were placed in 24-well plates. The integrity of the epithelial barrier was checked by measuring the transepithelial electrical resistance (TEER) using an epithelial voltohmmeter (Millicell-ERS STX-2 electrode system, Millipore) (22) and by means of a Dextran–Texas Red tracer solution (100 µg/ml; Invitrogen, Darmstadt, Germany) to assess the paracellular passage of low-molecular-weight molecules (23, 24). HIBCPP cells showed the common features of choroid plexus cells as determined by constitutive RNA expression of tight junction proteins (occludin, claudins 1-3, zonula occludens 1 [ZO-1]), E-cadherin, FOXJ1 (**Supplementary Figure 1**). For each TM experiment, 2 × 10^5^ B cells obtained from either healthy donors or MS patients were suspended in a 200 µl TM medium and transferred into the upper side of each insert (the basolateral side representing the lumen of the blood capillary). For B-cell activation, CD40/IgM (5 µg/ml) was added to the medium in some experiments. An amount of 500 µl TM medium, either alone or enriched with the B-cell chemoattractants CCL19, CCL20, CCL21, CXCL12, and CXCL13 in different concentrations and combinations with or without blocking monoclonal antibodies (mAbs) (purchased from R&D Systems, Minneapolis, MN, USA), was adjoined to the lower chamber (the apical side representing the CSF space). For some experiments, B cells were labeled with Cell Trace™ Calcein green (Invitrogen, Germany), according to the manufacturer’s instructions. The optimal TM time for assessing human B-cell migration was determined to 4 h in preliminary experiments (data not shown). After TM at 37°C, the filters were transferred onto a fresh 24-well plate and prepared for further analysis by either immunocytochemistry or transmission electron microscopy (TEM). Migrated B cells were collected from the lower chamber and immediately analyzed by flow cytometry and/or snap frozen and stored at -80°C before RNA extraction. Migration rates of total B cells were calculated from the number of B cells collected from the bottom chamber as percentage of the total B cells put into the upper chamber in the respective TM experiment. To assess for differences in migration activities of different B-cell subsets within the total B-cell population, specific chemotactic indices (CIs) for each subset were defined as the ratio of percentages of the distinct B-cell subpopulation in migrated B cells and in original B cells.

### Flow Cytometry

Multi-color flow cytometry experiments were performed to phenotypically characterize HIBCPP cells, to check the purity of isolated B cells, and to phenotypically and/or quantitatively analyze both migrated and non-migrated B cells after TM assays. Following an already published protocol (21, 28), PBMCs or isolated B lymphocytes were stained with mAbs specific for human B-cell surface markers (CD20-PerCP, CD27-PE, IgD-FITC, CD38-APC; purchased from BD Biosciences, Heidelberg, Germany) to quantitate CD20^+^ total B cells and to distinguish between CD20^+^CD27^-^IgD^+^ naïve, CD20^+^CD27^+^IgD^-^ class-switched memory (CSM), CD20^+^CD27^+^IgD^+^ non-switched memory (USM), CD20^+^CD27^-^IgD^-^ double-negative memory (DNM) and CD20^+^CD27^-^IgD^+^CD38^+^ transitional (TN) B-cell subtypes (**Supplementary Figure 2**). B cells were occasionally stained for different chemokine receptors or activation markers (CCR6, CXCR4, CXCR5, LFA-1, and VLA-4). A FACS Calibur™ cytometer plus CellQuest™ software (BD Biosciences) was used to measure and analyze all stained cells.

### RT^2^ Profiler PCR Arrays

The expression of 19 genes associated with B-cell activation and migration was evaluated after TM experiments in migrated and non-migrated B cells, respectively. We also screened the transcriptome of B cells isolated from parallel blood/CSF samples from a subcohort of ten MS patients. Total RNA from B cells was isolated and purified by the use of a RNeasy Micro kit (Qiagen, Hilden, Germany) and converted into cDNA with a RT^2^ First Strand kit (Qiagen). cDNA from each sample was pre-amplified using RT^2^ PreAMP PCR Mastermix and RT^2^ PreAMP Pathway Primer Mix and then added to a RT^2^ SYBR Green qPCR Master Mix on a custom array (Qiagen) designed to profile genes involved in the activation and migration process of human B lymphocytes. Each 96-well plate contained 4 sets of 19 specific genes of interest, two housekeeping genes for data normalization, one reverse-transcription (RT) control, one genomic DNA contamination control, and one positive PCR control (gene table is shown in **Supplementary Table 1**). After each run raw expression data were quantitatively analyzed by means of the ΔΔCt method utilizing the web-based software package (Qiagen), which automatically performed all the ΔΔCt-based fold-change calculations from the uploaded raw Ct data. The x-fold change was calculated as the normalized gene expression (2^–^ΔΔ^Ct^) between the chosen sample groups. The results are presented in tabular format.

### Quantitative Real-Time PCR Analysis

Quantitative real-time PCR for determination of constitutive gene expression in HIBCPP cells was performed using the SuperScript III Platinum SYBR Green One-Step qRT-PCR Kit and an Applied Biosystems 7500 Fast Dx Real-Time PCR Instrument (both Life Technologies GmbH, Darmstadt, Germany) following the manufacturer’s instructions. PCR primers are listed in **Supplementary Table 2**.

### PCR-Based High-Resolution Capillary Electrophoresis

PCR-based high-resolution capillary electrophoresis for determination of the IgH CDR3 locus was performed as described previously (29). In short, PCR was performed with 50 ng of B-cell cDNA using VH-Fr3A (5´-ACACGGC(C/T)(G/C)TGTATTACTGT) and LJH (5´-TGAGGAGACCGGTGACC) primers. VH-Fr3A-primer was labeled with 6-carboxyfluorescein for subsequent automated fluorescent fragment analysis. Therefore, 3 µl of PCR products were mixed with 0.5 µl of internal size standard GeneScan 350-TAMRA (Applied Biosystems, Weiterstadt, Germany) in 20 µl formamide, denaturated for 2 min at 95°C and then subjected to a laser-induced fluorescent capillary electrophoresis system (ABI Prism 310, Applied Biosystems). GeneScan Analysis Software (Applied Biosystems) was used for quantification and size determination.

### Immunocytochemistry

TM transwell filters were fixed/permeabilized, incubated overnight with mAbs specific for tight junctions, cytoskeleton, and B-cell markers, then exposed to secondary mAbs, and finally analyzed using a Zeiss Apotome microscope^®^ as described elsewhere (24).

### Transmission Electron Microscopy

TEM analysis was performed in collaboration with Dr. Ingrid Haußer, Electron Microscopy Core Facility (EMCF), Heidelberg University. In short, TM transwell filters were fixed in 2.5% glutaraldehyde, postfixed with 1% osmium tetroxide, cut into strips, dehydrated, and embedded in epoxy resin. 60 nm ultrathin sections were cut with an ultramicrotome, contrasted with uranyl acetate/lead citrate, and analyzed with a TEM JEM140 equipped with a digital camera (TVIPS F420).

### Statistical Analysis

Statistical analysis was performed with Excel (Microsoft) and SPSS (SPSS Inc., Chicago, IL, USA). For analysis of multiple groups one-way ANOVA was performed and corrected by post Hoc Tukey test. Mann–Whitney U-tests or Wilcoxon tests were used to compare non-normally distributed paired and unpaired samples. Q-Q plots were used to check for the normality assumption of a data set. *P*-values of < 0.05 < 0.01, or < 0.001 were considered to indicate significant, highly significant or extremely significant statistically difference. All data represent mean values ± standard deviation (SD).

## Results

### Transmigration Rates of Human B Lymphocytes Across Human Choroid Plexus Papilloma

**Figure 2:**
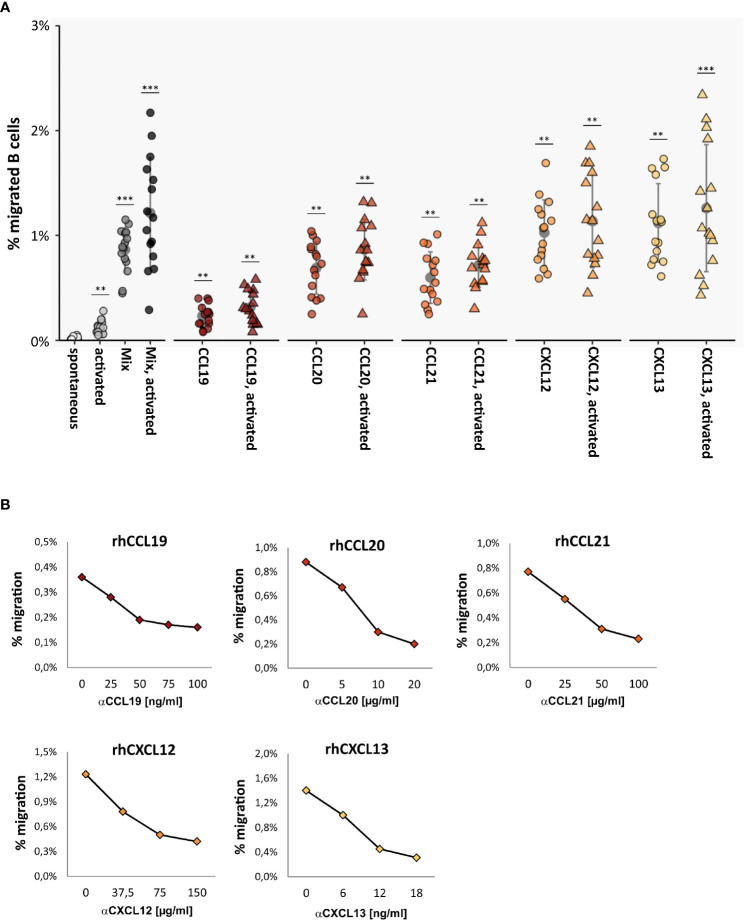
Chemokine-induced transmigration of normal B cells across the HIBCPP monolayer. **(A)** TM rates of human total CD19^+^ B cells obtained from 15 healthy donors in the absence and presence of B-cell stimuli and chemokines. When various B-cell-specific chemokines were added alone or in combination to the lower chamber, TM rates multiplied by approximately 50-fold, with CXCL12 and CXCL13 being most effective. TM rates were further enhanced (approximately 1.5-fold) when B cells were activated with anti-CD40 & IgM during chemokine-triggered migration. All experiments were carried out in triplicates. Dots represent data per individuals. Means are indicated (grey circles). Error bars denote SD. Asterices indicate statistical significances compared with spontaneous TM (***P < 0.001, **P < 0.01; one-way ANOVA plus post Hoc Tukey test). Chemokine concentrations: CCL19 [500 ng/ml], CCL20 [100 ng/ml], CCL21 [120 ng/ml], CXCL12 [100 ng/ml], and CXCL13 [1 µg/ml]. “Mix” = all chemokines; “activated” = CD40 mAb [5 µg/ml] & IgM [12.5 µg/ml]. **(B)** Blocking of chemotactic activities by neutralizing mAbs. TM rates of CD40/IgM-stimulated B cells towards indicated chemokines in the presence of increasing concentrations of neutralizing mAbs. All experiments were carried out in triplicates.

Total CD19^+^ B cells were immunomagnetically isolated from blood samples of 60 healthy donors and immediately assessed in TM assays. Spontaneous TM rates of unstimulated B cells across HIBCPP were low, with only sporadic B cells detectable in the bottom chamber [average 0.02 ± 0.01% (migrated B cells of all B cells)]. When various B-cell-specific chemokines were added alone or in combination to the lower chamber, TM rates multiplied by approximately 50-fold, with CXCL12 (1.03 ± 0.30%, *p* < 0.001) and CXCL13 (1.12 ± 0.36%, *p* < 0.001) being most effective (**Figure 2A**).

We next assessed the effect of B-cell activation on chemokine-induced transmigration. In preliminary experiments, stimulation of freshly isolated human B cells with anti-CD40/IgM turned out to be the most effective mode to achieve fast B-cell activation (data not shown). We therefore used this approach for additional stimulation of B cells during chemokine-induced transmigration, resulting in moderately enhanced TM rates (approximately 1.5-fold) as shown in **Figure 2A**.

The specificity of the chemotactic effects mediated by each chemoattractant was confirmed by blocking experiments using different concentrations of neutralizing mAbs specific for each chemokine (**Figure 2B**). As determined by TEER-measurement and by detection of the paracellular Dextran-flux, the barrier integrity of the HIBCPP layer was not affected by B-cell transmigration under different conditions tested here (**Supplementary Figure 3**).

### Migrated B Cells Predominantly Exhibit a Class-Switched Memory Phenotype

**Figure 3:**
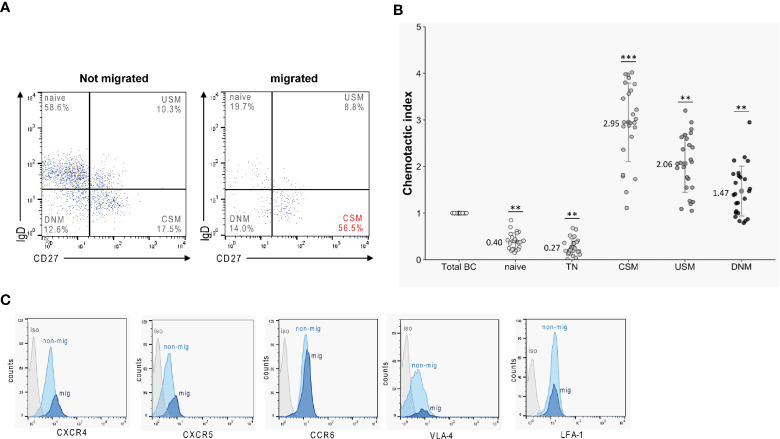
Transmigration rates of B-cell subsets. B cells obtained from healthy donors (*n* = 25) were tested in TM assays against a mix of CXCL12 and CXCL13. Migrated and non-migrated cells were then phenotypically characterized by flow cytometry. **(A)** Migrated cells were highly enriched for class-switched memory B cells (CSM). **(B)** Superior chemotactic activity (CI) of memory B-cell subsets (CSM, class-switched memory; USM, non-switched memory; DNM, double negative memory) versus naïve subtypes (TN, transitional). All experiments were carried out in triplicates. Dots represent data per individuals. Dots represent data per individuals. Means are indicated (grey circles). Error bars denote SD. Asterices indicate statistical significances compared with spontaneous TM (***P < 0.001, **P < 0.01; one-way ANOVA plus post Hoc Tukey test). **(C)** Surface expression of CXCR4, CXCR5, CCR6, VLA-4 and LFA-1 on migrated (mig, light blue) and non-migrated (non-mig, light blue) CSM B cells obtained from healthy donors (*n* = 10) as determined by flow cytometry. (Isotype control = iso, in gray).

Before and after TM against a mix of CXCL12 and CXCL13, B lymphocytes—isolated from blood samples obtained from 25 HC —were phenotypically characterized by flow cytometry to identify B-cell subtypes. Migrated cells were highly enriched in CD27^+^IgD^-^ class-switched memory B cells (CSM) (**Figure 3A**). Accordingly, when calculating chemotactic indices (CI) for each subset - defined as the ratio of percentages of the distinct B-cell subsets in migrated B cells versus those in original B cells - we found the highest chemotactic activities for CSM B cells (CI: 2.95 ± 0.56) (**Figure 3B**). CIs of the remaining memory B-cell subsets (CD27^+^IgD^+^ USM: 2.06 ± 0.60; CD27^-^IgD^-^ DNM: 1.47 ± 0.75) also ranged >1, indicating enhanced chemotaxis. In contrast, low CIs < 1 were calculated for all naïve subtypes (CD27^-^IgD^+^ naïve: 0.40 ± 0.13; CD27^-^IgD^+^CD38^+^ TN: 0.27 ± 0.15) (**Figure 3B**). Staining of occasional transmigrated and non-transmigrated B-cell preparations for receptors involved in immune cell trafficking (CXCR4, CXCR5, CCR6) and cell adhesion (LFA-1, VLA-4) (n = 10), revealed enhanced expression of CXCR4 [135 ± 14 (mean fluorescence intensity, MFI)] and CXCR5 [96 ± 7 (MFI)] on migrated CSM B cells when compared to cells that had not migrated [CXCR4: 112 ± 16 (MFI), *p* < 0.05; CXCR5: 81 ± 7 (MFI), *p* < 0.05 (**Figure 3C**, **Supplementary Figure 4**)].

### Chemotactic Activities Are More Potent in Multiple Sclerosis- Versus Healthy Control-Derived Class-Switched Memory B Cells

**Figure 4:**
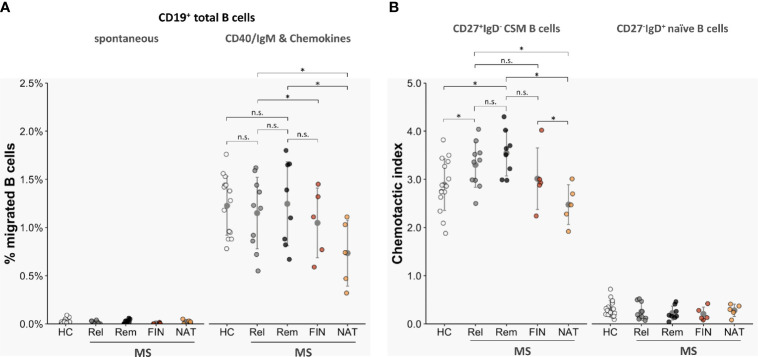
Transmigration of MS-derived B-cell subsets across HIBCPP cells. Chemotactic activities of CD19^+^ total B cells, CD19^+^CD27^+^IgD^-^ class-switched memory B cells (CSM), and CD19^+^CD27^-^IgD^+^ naive B cells isolated from peripheral blood of 15 healthy donors (HC) and 28 MS patients (untreated, in relapse: *n* = 10 (Rel); untreated, in remission: *n* = 8 (Rem); fingolimod-treated: *n* = 5 (FIN); natalizumab-treated: *n* = 5 (NAT)). **(A)** TM rates of total CD19^+^ B cells derived from treatment-naïve MS patients were not altered compared with controls, but where significantly lower when obtained from NAT-treated patients. **(B)** Overall, chemotactic indices (CI) of CSM B cells were clearly higher than those of CD27^-^ naïve B cells. CIs where further enhanced for CSM B cells derived from non-treated MS but were significantly lower when tested with CSM B cells obtained from NAT-treated individuals. All experiments were carried out in triplicates. Dots represent data per individuals. Means are indicated (grey circles). Error bars denote SD. Asterices indicate statistical significances compared with spontaneous TM (*P < 0.05, n.s., not significant; one-way ANOVA plus post Hoc Tukey test).

We next compared transmigration rates of total CD19^+^ B cells as well as of CD19^+^CD27^+^IgD^-^ class-switched memory B cells and CD19^+^CD27^-^ naive B cells isolated from blood samples of 18 untreated MS patients (n = 10 in acute relapse; n = 8 in clinical remission) and 15 age- and sex-matched HC. Unstimulated CD19^+^ B cells from the two cohorts exhibited similarly low spontaneous TM rates (HC: 0.02 ± 0.01%; MS, relapse: 0.01 ± 0.01%; MS, remission: 0.03 ± 0.02% [migrated B cells as proportion of all B cells]; non-significant with *p* > 0.05 each) (**Figure 4A**). TM rates of total B cells activated by anti-CD40/IgM and tested against a chemokine mix (CCL19, CCL20, CCL21, CXCL12, CXCL13) were overall heightened but also did not differ between patient-derived and donor-derived B cells (HC 1.23 ± 0.22%; MS, relapse: 1.15 ± 0.36%; MS, remission: 1.25 ± 0.39%; non-significant with *p* > 0.05 each) (**Figure 4A**). When testing purified B cell subsets, however, CD27^+^IgD^-^ class-switched memory B cells (CSM) from MS patients displayed more pronounced chemotactic activity than those from control donors [HC: 2.89 ± 0.31 (chemotactic index, CI); MS, relapse: 3.30 ± 0.28; MS, remission: 2.55 ± 0.30; *p* < 0.05 each] (**Figure 4B**), while trafficking of CD27^-^IgD^+^ naïve B-cell subtypes across HIBCPP was unequivocally very low in both cohorts. Altogether, TM rates did not differ significantly between B cells and B-cell subsets obtained from MS patients with active disease or those in clinical remission and were not correlated with patients Q_Alb_ (data not shown).

### Altered Trafficking of Memory B Cells Exposed to Disease-Modifying Treatment

Additional data were gained from five fingolimod-treated and five natalizumab-treated MS patients. As expected and previously described (21), B-cell distribution in these patients showed characteristic changes and was reciprocally altered: while fingolimod prompted a clear reduction circulating in memory B cells, natalizumab provoked a significant expansion of these subtypes (**Supplementary Table 3**). Notably, as depicted in an independent cohort of patients switching from NAT to a different immunomodulatory drug the very same CSM B cells vanished from peripheral blood and became intraindividually enriched in the CSF of those individuals who experienced a relapse during the washout period (**Supplementary Figure 5**).

Trafficking of fingolimod-exposed total B cells [1.05 ± 0.30% (migrated B cells as proportion of all B cells)] and CD27^+^IgD^-^ CSM B cells [3.01 ± 0.36 (chemotactic index, CI)] stimulated and tested against the chemokine mix, trended to be lower than in non-treated patients, although the difference did not attain statistical significance (**Figures 4A, B**). In contrast, total B cells from natalizumab-treated patients displayed lower B-cell trafficking (0.73 ± 0.28%) when compared to non-treated patients with acute relapse (*p* < 0.05) or non-treated patients in clinical remission (*p* < 0.05): Notably, the migratory capacity of CSM B cells was even more downscaled [2.48 ± 0.27 (CI), *p* < 0.05; **Figures 4A, B**].

### Human B Cell Trafficking Across the Choroid Plexus Epithelium Display Distinct Gene Expression Profiles

**Figure 5:**
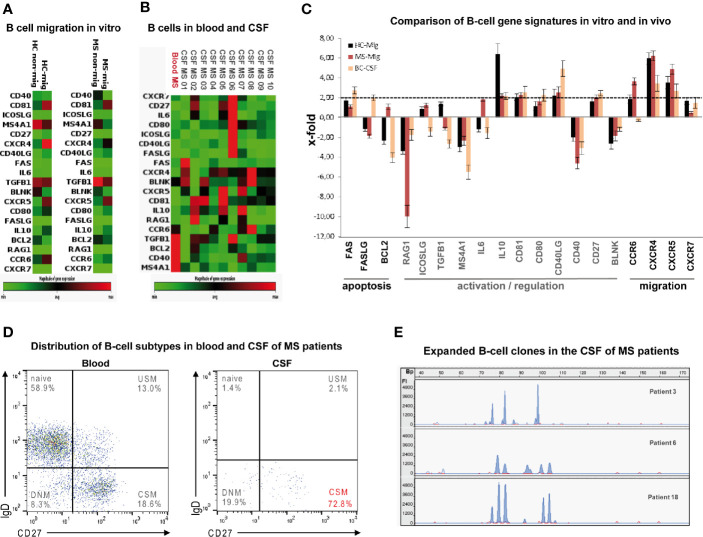
Phenotypes and gene signatures of B cells migrated *in vitro* compared to intrathecal B cells from MS patients. Heat map of customized PCR array showing differential expression of 19 genes involved in apoptosis, activation/regulation and migration in **(A)** *in vitro* migrated against a mix of CXCL12 and -13 (mig = average expression of 15 samples) and non-migrated (non-mig = average expression of 15 samples) blood-derived B cells obtained from 15 healthy donors and ten MS patients and in **(B)** CSF-derived (patient 01–10) and blood-derived (“Blood MS” = average expression of 10 samples). B cells obtained from 10 MS patients with active disease as determined by PCR array. Green = low expression; red = high expression. **(C)** Comparison of B-cell gene signatures *in vitro* and *in vivo*: Bars denote means of x-fold changes in gene expression in B cells obtained from 15 HC (mig-HC, black) and from ten MS patients (mig-MS, red) that had migrated through the HIBCPP layer as well as in CSF-derived B cells obtained from 10 MS patients with active disease (CSF-MS, yellow). Error bars denote SD. **(D)** Flow cytometry of CSF-derived total CD19^+^ B cells obtained from 10 MS-patients with active disease shows predominance of class-switched memory cells (CSM) (USM: non-switched memory, DNM: double negative memory). **(E)** Electrophoretic gene scanning profiles of fluorescent IgH CD3 PCR amplicons generated from CSF-derived total CD19^+^ B cells obtained from three MS-patients with active disease (patients 3, 6, and 18) depict 4-6 expanded B-cell clones. Relative fluorescence intensities (FI, y-axis) are plotted against amplicon size in base pairs (Bp, x-axis).

To assess the gene expression profiles of B lymphocytes that migrated through the choroid plexus epithelium, total CD19^+^ B cells obtained from ten MS patients with clinically active disease and 15 normal donors were tested in TM assays against a mix of CXCL12 and CXCL13. When comparing gene expressing profiles of migrated with non-migrated B cells, we found upregulation of several genes involved in both B-cell activation and trafficking, as for example CD81, CD40L, CXCR4, and CXCR5 in both cohorts (**Figures 5A, B** and **Supplementary Table 4**).

To extend the findings to the *in vivo* situation we additionally compared gene expressing profiles of paired blood- and CSF-derived total B cells in a subcohort of 10 MS patients with clinically active disease. As a striking observation, very similar to B cells migrating though HIBCPP cells, intrathecal B cells predominantly displayed a CSM phenotype (**Figure 5C**) along with upregulated CXCR4 and CXCR5 mRNA expression confirming an active migratory state (**Figures 5A, B** and **Supplementary Table 4**). Three samples (patients 3, 6, 18) of CSF-derived B-cells were randomly checked by IgH-CD3-PCR (29) and were found to be oligoclonal as depicted by 4–6 expanded B-cell clones respectively (**Figure 5D**).

### Human B Cells Use Both Para- and Transcellular Diapedesis for Transmigration Through the Choroid Plexus Epithelium

**Figure 6:**
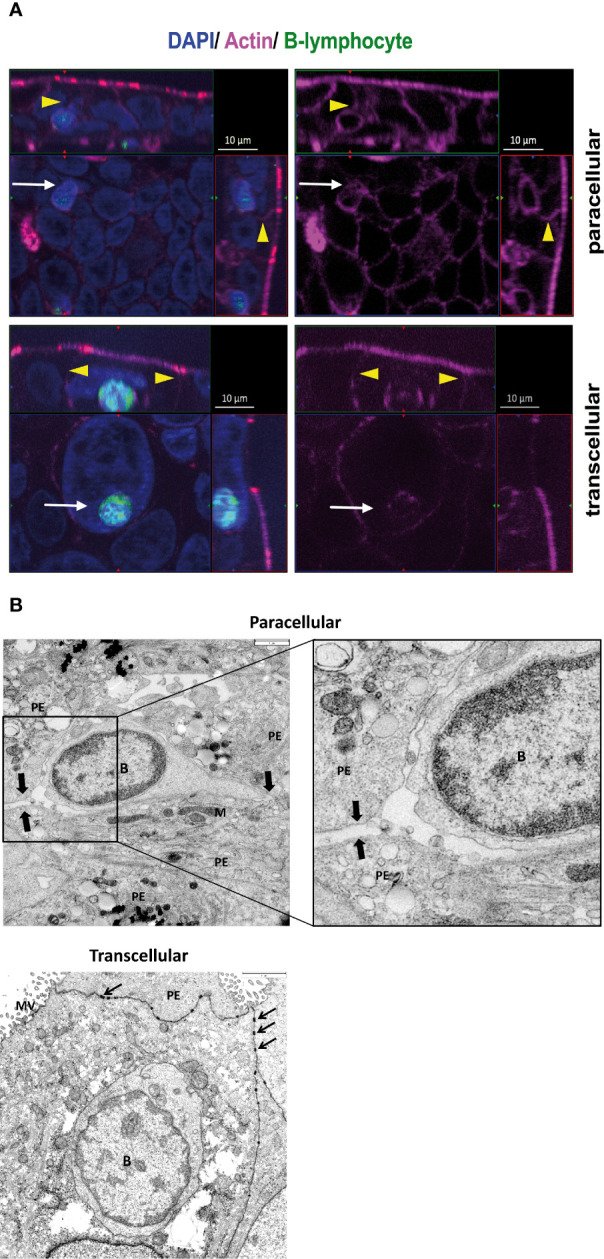
Human B cells use both para- and transcellular diapedesis for transmigration through the choroid plexus epithelium. **(A)** Immunofluorescence analysis of B-cell migration across HIBCPP cells. Diapedesis of total CD19^+^ B cells obtained from healthy donors (*n* = 15) was analyzed using immunofluorescent staining. After 4 h TM against a mix of CXCL12- and 13 the filters for nuclei were stained with DAPI (shown here in blue) and actin skeleton with phalloidin (shown here in pink). B lymphocytes (shown here in green) were labeled with Cell Trace™ Calcein green before starting the migration. Z-stack images were taken using an ApoTome (Zeiss) and represented through display of a cross section through the z-plane of multiple slices in combination with a two-dimensional view from the top of the cell layer. The polarized cells are oriented with the apical side pointing upwards or to the right and the basolateral side downwards or to the left. Localization of migrating B cells in relation to the actin skeleton is indicated by yellow arrowheads. The upper immunofluorescence image depicts a single B cell (white arrow) located in the intercellular space between several HIBCPP cells (blue) and thus, uses a paracellular route to cross the epithelium. The intercellular borders (yellow arrowheads) are visualized in the actin staining and can be clearly seen above the migrating B cell (white arrow). The lower image maps a single B cell (white arrow) located inside a HIBCPP cell, at a clear distance from the cell borders (yellow arrowheads), which is in favor of transcellular diapedesis. Images shown are representative examples of multiple stainings taken from five independent experiments each performed in triplicate. **(B)** For deeper analysis of B-cell diapedesis across HIBCPP cells total CD19^+^ B cells obtained from healthy donors (*n* = 10) were analyzed using Transmission electron microscopy (TEM). After 4 h TM the filters were, therefore, fixed in 2.5% glutaraldehyde, postfixed with 1% osmium tetroxide, cut into strips, dehydrated, and embedded in epoxy resin. 60 nm ultrathin sections were cut with an ultramicrotome, contrasted with uranyl acetate/lead citrate, and analyzed with a TEM JEM140 equipped with a digital camera (TVIPS F420). Top: paracellular B-cell migration across HIBCPP clearly seen by a continuous intercellular route indicated by arrows. Higher magnification shows the migrating rear part of a B cell, moving clearly intercellulary between epithelial cells. Bottom: transcellular B-cell migration across HIBCPP. One B cell is located inside an epithelial cell and migrates at a clear distance from the cell border. Scale bars: 3 µm. PE, plexus epithelial cell; B, B lymphocyte nucleus; arrows, desmosomes; M, mitochondrium; MV, microvilli.

Diapedesis routes of B cells through HIBCPP cells were analyzed by both immunocytochemistry and TEM. Immunofluorescence analysis revealed that activated B cells migrate *via* both the para- and transcellular routes across HIBCPP cells. In **Figure 6A**, the upper immunofluorescence image depicts a single B-cell (green, white arrow) located in the intercellular space between several HIBCPP cells (blue) and thus, uses a paracellular route to cross the epithelium. The intercellular borders are visualized in the actin staining (yellow arrowheads) and can be clearly seen above the migrating B-cell (white arrows). The lower image maps a single B-cell (green, white arrow) located inside a HIBCPP cell, at a clear distance from the cell borders (yellow arrowheads), reaching the CSF space by transcellular diapedesis. To confirm and extend the immunocytochemistry data we performed TEM analysis. **Figure 6B** top shows a single B-cell during paracellular crossing of HIBCPP cells. Higher magnification shows the migrating rear part of a B-cell, moving clearly intercellulary between epithelial cells. **Figure 6B** bottom shows a single B-cell trafficking *via* the transcellular pathway with the B-cell totally surrounded by cytoplasm of the epithelial cell. Again, this prototype cell is located inside an epithelial cell and migrates at a clear distance from the cell border. Thus, B cells can migrate *via* either the paracellular or the transcellular route through the epithelial BCSFB.

## Discussion

The migratory pathways and trafficking mechanisms that allow peripheral immune cells to enter the CSF across the choroid plexus epithelium constituting the anatomical basis of the BCSFB are incompletely understood (9, 30–32). The BSCFB, together with the BBB and the BMB, tightly controls the influx of cells and macromolecules into the CNS and, when dysfunctional, might promote and sustain chronic inflammation such as occurs in MS, a prototypical autoimmune disorder of the CNS. This may be particularly true for B cells, which are virtually absent in normal CSF, yet in patients with MS accumulate predominantly as CSM phenotypes in the CSF compartment, as we and others have shown previously (17–21). Since the role of the BSCFB in governing migration of B cells into the CNS is thus far undefined, we performed a detailed study to assess the interaction between human B lymphocytes and the choroid plexus epithelium and to define its impact on potentially harmful B-cell responses in MS. For this purpose, we adapted an *in vitro* model based on HIBCPP cells, that shows all characteristic features of choroid plexus epithelium—the morphological correlate of the BCSFB—including formation of tight junctions, development of a high transepithelial electrical resistance (TEER), expression of minor permeability for macromolecules, apical/basolateral polarity, and expression of characteristic transporter molecules (22, 32, 33). This model, in the past, has been successfully applied to assess leukocyte trafficking through the choroidal epithelium and to analyze barrier alterations and specific immune responses during diapedesis (23, 24, 34, 35).

We found that B cells barely traffic across HIBCPP cells spontaneously and—as a prerequisite to acquisition of migratory properties - require a B-cell-friendly environment provided by B-cell-attracting or homeostatic chemokines and, in addition, activation *via* the B-cell receptor to achieve the most efficient diapedesis. Hence, migration through the BCSFB appears to imply the presence of a preactivated B-cell state. This observation is in line with the sparseness of this subset in normal CSF (17) and also corroborates the view that in patients with acute MS relapses, immune cells are activated in the periphery and subsequently cross barriers to enter CNS compartments including the CSF (6, 7). Of note, whereas B cells obtained from MS patients did not exhibit any greater tendency overall to migrate across HIBCPP cells and did not respond differently to chemotaxis, prestimulated and non-stimulated memory B-cell subsets with a marked predominance of isotope-switched phenotypes from both patients with active and inactive disease displayed greater trafficking properties in response to B-cell-specific chemokines than their counterparts from healthy donors, suggesting that in MS antigen-experienced CSM B cells are particularly endowed with more efficient trafficking skills through the choroid plexus. In accordance with this assumption, patient-derived B cells are polarized towards a proinflammatory phenotype (20, 30, 36), and peripheral memory subsets appear to be redirected to the intrathecal space, a process that conditions their local conversion into antibody-secreting effector cells. Hence, as we have shown in a previous study, relapsing and to a lesser extent also stable MS coincide with a compartmentalized shift in B-cell homeostasis consisting of contracted proportions of memory phenotypes among total peripheral blood B cells along with a reciprocal expansion of CSM B cells in intraindividual CSF specimens (21). Importantly, such crossover changes in the CSF were linked to intrathecal frequencies of effector plasma blasts/plasma cells and intrathecal concentrations of CXCL13, the homeostatic and B-cell-attracting cytokine that—in this study—prompted the most abundant B-cell migration (37, 38). Of note, prolonged disease modifying treatment with natalizumab—and to a lesser, yet not significant degree with fingolimod—prompted decreased B-cell trafficking across HIBCPP cells. Whole B cells and purified CSM B cells from natalizumab-treated patients—supposedly by blocking of VLA-4 expressed on the surface B cells—moved through HIBCPP cells less efficiently versus those from both fingolimod-exposed patients and normal donors. Migration rates were similarly decreased when using total or separated CD27^+^ B cells and hence, downscaled trafficking occurred independent from drug-induced changes in B-cell homeostasis. Despite the overall low number of drug-exposed patients assessed, these results strengthen the role of the BSCFB in promoting B-cell entry into the CNS and highlights the observation that B-cell VLA-4 deficiency impedes susceptibility of CNS autoimmunity in mice (39). The data are also in line with the notion that fingolimod is less potent than natalizumab in keeping B cells sequestered from the CSF (37, 38). Altogether, these findings indirectly support the concept that B-cell diapedesis at the choroid plexus epithelium is mainly promoted by the interplay of circulating CSM B cells in the periphery and the B-cell-friendly milieu in the CSF. Accordingly, when comparing migrated with non-migrated B cells on a transcriptional level, we found enhanced expression of several genes involved in B-cell activation and trafficking corroborating the fact that preferentially B cells with higher activation status and upregulated chemokine receptors are able to penetrate the BLS. Of note, intrathecal B cells from treatment-naïve MS patients with clinically active disease, displayed an almost similar migratory phenotype as depicted by co-expression of CD27 and upregulation of CXCR4 and CXCR5 genes, thus strengthening the fundamental interplay between CSM memory phenotypes and the CXCL-12/-13 axis for B-cell penetration through the BCSFB. Notably, the tremendous clinical importance of such “migratory” B cells is strongly underlined by our finding in a restricted number of patients, that CD27^+^ isotype switched B cell phenotypes change compartments, i.e. disappear in blood and accumulate intrathecally, when MS activity recurs after withdrawal from NAT therapy, thus qualifying this subset as an attractive biomarker of upcoming MS reactivation. The harmful role of CSM-B cells is further supported by the preliminary observation that their frequencies in CSF correlate with MS disease activity and disability (unpublished finding), How B cells attracted by intrathecal chemokines CXCL-12 and -13 achieve diapedesis across the BSCFB is incompletely understood. Whether B cells reside directly in the human and murine inner stroma of the naive choroid plexus, as reported for T cells (8, 40), remains to be determined. However, similar to other immune cells, B cells are not restricted from leaving the non-tight-junction-connected choroidal microvessels and, in addition, as shown here for migratory B cells express leukocyte-function-associated antigen 1 (LFA-1), the ligand of ICAM-1 as well as VLA-4, the ligand of VCAM-1 and fibronectin. These constituents are required for immune cell trafficking and in part have been found to assist B cells in migrating through human brain-derived endothelial cells modelling the BBB (41). The relevance of the VLA-4/VCAM-1 axis for B-cell trafficking into the CSF is further underlined by the reduced trafficking capacities across the choroid plexus displayed by B cells obtained from MS patients exposed to the VLA-4 blocking agent natalizumab as shown in this study and the overall efficiency of natalizumab in the treatment of MS (38, 39). The reduced expression of the tight junction protein claudin-3 as denoted histopathologically in choroid plexus epithelial cells from MS patients might further facilitate the entry of B cells and other immune cells across the interface (42). Detailed assessment of trafficking routes by immunocytochemistry and TEM revealed that B-cell migration from the blood can occur by either paracellular or transcellular diapedesis of HIBCPP cells in the presence of an intact barrier function as assessed by paracellular permeability and transepithelial electrical resistance. The use of both pathways appears to be a universal feature of leukocytes: in the same BCSFB model, polymorphonuclear neutrophils, monocytes, and T-cells also simultaneously travel towards the apical border of HIBCPP cells by crossing either between or directly across epithelial cells without evidence of barrier dysfunction (24, 35). Moreover, transepithelial migration of leukocytes appears to be mechanistically shared between the BCSFB and the BBB, where moving immune cells also overcome the tight junction-sealed endothelium by both paracellular and transcellular transit (43).

## Conclusions

**Figure 7:**
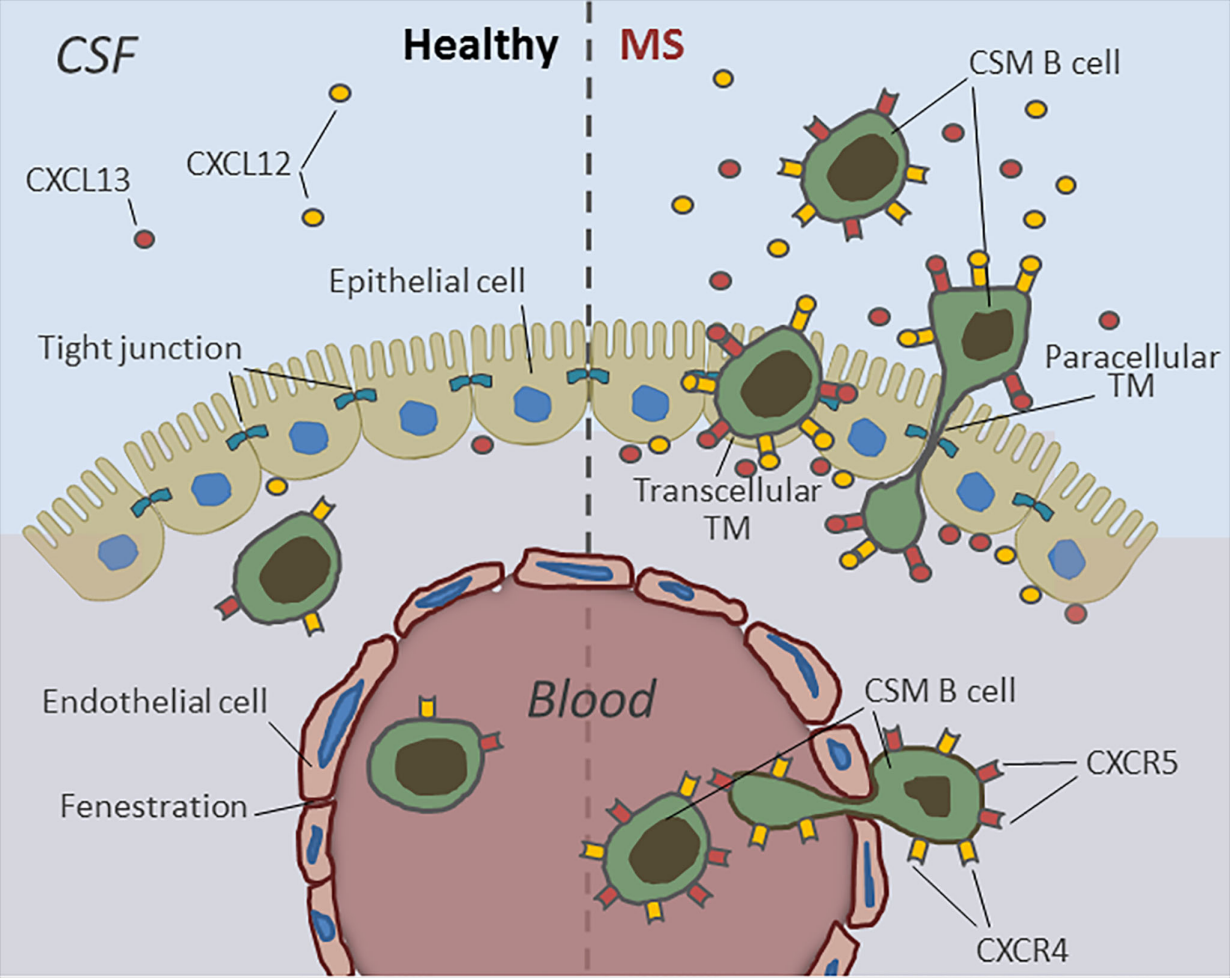
Human B-cell migration through the blood–CSF-barrier in health and during active MS. B lymphocytes can easily pass the fenestrated blood vessels within the choroid plexus parenchyma but rarely enter the CSF due to the barrier properties of CP epithelial cells. During active MS the choroid plexus epithelium becomes permeable for pre-activated, CXCR4- and CXCR5 expressing antigen-experienced class-switched memory B cells (CSM). The transmigration of CSM B cells into the CSF is mediated by high intrathecal concentrations of B-cell specific chemokines CXCL12- and -13 and occurs both by transcellular and paracellular diapedesis.

Taken together, these data suggest a model whereby abnormalities in the immunologic milieu surrounding the choroid plexus epithelium on both the basal and apical cell borders in the context of chronic neuroinflammation are likely to amplify the permissiveness of the choroid plexus for B-cell entry *in vivo* and imply that the BSCFB substantially accounts for the selective enrichment of potentially harmful CSM B cells in the CSF of MS patients (**Figure 7**). Moreover, this gate might—in addition to facilitate aberrant B-cell accumulation in the CSF and CNS of MS patients—at least in part, contribute to the bidirectional cell exchange that has been postulated to explain why, in MS, the same clonally expanded B cells can be found within different CNS compartments (31, 44). Counteracting such dialog is therapeutically highly relevant, the more so as a central role of memory B cells in MS pathophysiology is supported by the fact that many disease-modifying drugs, including alemtuzumab, rituximab, fingolimod, and natalizumab, destroy or functionally deplete memory B-cell activity, while atacicept—an antagonist to B-cell-activating factor (BAFF) and A-proliferation-inducing ligand (APRIL) and or infliximab—an antagonist to TNF-alpha—which both stimulate memory B cells, were shown to worsen the disease (45).

## Data Availability Statement

The datasets presented in this study can be found in online repositories. The names of the repository/repositories and accession number(s) can be found in the article/**Supplementary Material**.

## Ethics Statement

The study design was approved by the ethics committee of the University Hospital Heidelberg. Written informed consent was obtained from all study participants.

## Author Contributions

BW and JH conceptualized and designed the study. JH, HR, SF, SL, CW, LC, and CS-G acquired the data. JH, BW, HR, ChS, and TT analysed and interpreted the data. JH and BW drafted the manuscript. BW, JH, SJ, TT, ChS, and HS critically revised the manuscript for important intellectual content. JH performed the statistical analysis. HI, HS, and BW provided administrative, technical, and material support. BW and JH supervised the study. All authors contributed to the article and approved the submitted version.

## Funding

This work was supported by grants from the German Ministry for Education and Research {BMBF; German Competence Network Multiple Sclerosis [KKNMS, Research Consortium 3 “Prognostic and treatment markers”: ReboundMS, 01GI11603 (Wildemann)]} the Klaus Tschira Foundation, and Novartis. We also acknowledge financial support by Deutsche Forschungsgemeinschaft within the funding program Open Access Publishing, by the Baden-Württemberg Ministry of Science, Research and the Arts and by Ruprecht-Karls-Universität Heidelberg. None of the funding sources had a role in the study design; collection, analysis, and interpretation of data; writing of the report; or the decision to submit the paper for publication.

## Conflict of Interest

BW has received research grants and/or honoria from Alexion, Merck Serono, Biogen, Teva, Novartis, Sanofi Genzyme, Bayer Healthcare, and research grants from the Dietmar Hopp Foundation, the Klaus Tschira Foundation and the Deutsche Forschungsgemeinschaft (DFG).

The remaining authors declare that the research was conducted in the absence of any commercial or financial relationships that could be construed as a potential conflict of interest.

## References

[1] Man S Ubogu EE Ransohoff RM Inflammatory cell migration into the central nervous system: a few new twists on an old tale. Brain Pathol (2007) 17:243–50. 10.1111/j.1750-3639.2007.00067.x PMC8095646 17388955

[2] Pendergrast CT Anderton SM Immune cell entry to central nervous system – current understanding and prospective therapeutic targets. Endocrine Metab Immune Disord Drug Targets (2009) 9:315–27. 10.2174/187153009789839219 20028334

[3] Ransohoff RM Engelhardt B The anatomical and cellular basis of immune surveillance in the central nervous system. Nat Rev Immunol (2012) 12:623–35. 10.1038/nri3265 22903150

[4] Shechter R London A Schwartz M Orchestrated leukocyte recruitment to immune-privileged sites: absolute barriers versus educational gates. Nat Rev Immunol (2013) 13:206–18. 10.1038/nri3391 23435332

[5] Reboldi A Coisne C Baumjohann D Benvenuto F Bottinelli D Lira S C-C chemokine receptor 6-regulated entry of TH-17 cells into the CNS through the choroid plexus is required for the initiation of EAE. Nat Immunol (2009) 10:14–23. 10.1038/ni.1716 19305396

[6] Compston A Cole A Multiple sclerosis. Lancet (2008) 372:1502–17. 10.1016/S0140-6736(08)61620-7 18970977

[7] Comabella M Khoury SJ Immunopathogenesis of multiple sclerosis. Clin Immunol (2012) 142:2–8. 10.1016/j.clim.2011.03.004 21458377

[8] Kivisäkk P Mahad DJ Callahan MK Trebst C Tucky B Wei T Human cerebrospinal fluid central memory CD4+ T cells: evidence for trafficking through choroid plexus and meninges via P-selectin. Proc Natl Acad Sci USA (2013) 100:8389–94. 10.1073/pnas.1433000100 PMC166239 12829791

[9] Strazielle N Creidy R Malcus C Boucraut J Ghersi-Egea JF T-Lymphocytes Traffic into the Brain across the Blood-CSF Barrier: Evidence Using a Reconstituted Choroid Plexus Epithelium. PloS One (2016) 11:e0150945. 10.1371/journal.pone.0150945 26942913 PMC4778949

[10] Hauser SL Waubant E Arnold DL Vollmer T Antel J Fox RJ B-cell depletion with rituximab in relapsing-remitting multiple sclerosis. N Engl J Med (2008) 358:676–88. 10.1056/NEJMoa0706383 18272891

[11] Hauser SL Bar-Or A Comi G Giovannoni G Hartung HP Hemmer B Ocrelizumab versus Interferon Beta-1a in Relapsing Multiple Sclerosis. N Engl J Med (2017) 379:221–34. 10.1056/NEJMoa1601277 28002679

[12] Montalban X Hauser SL Kappos L Arnold DL Bar-Or A Comi G Ocrelizumab versus Placebo in Primary Progressive Multiple Sclerosis. N Engl J Med (2017) 378:209–20. 10.1056/NEJMoa1606468 28002688

[13] Lucchinetti C Brück W Parisi J Scheithauer B Rodriguez M Lassmann H Heterogeneity of multiple sclerosis lesions: implications for the pathogenesis of demyelination. Ann Neurol (2000) 47:707–17. 10.1002/1531-8249(200006)47:6<707::AID-ANA3>3.0.CO;2-Q 10852536

[14] Meinl E Krumbholz M Hohlfeld R B lineage cells in the inflammatory central nervous system environment: migration, maintenance, local antibody production, and therapeutic modulation. Ann Neurol (2006) 59:880–92. 10.1002/ana.20890 16718690

[15] von Büdingen HC Bar-Or A Zamvil SS B cells in multiple sclerosis: connecting the dots. Curr Opin Immunol (2011) 23:713–20. 10.1016/j.coi.2011.09.003 PMC4188435 21983151

[16] Srivastava R Aslam M Kalluri SR Schirmer L Buck D Tackenberg B Potassium channel KIR4.1 as an immune target in multiple sclerosis. N Engl J Med (2012) 367:115–23. 10.1056/NEJMoa1110740 PMC5131800 22784115

[17] Cepok S Rosche B Grummel V Vogel F Zhou D Sayn J Short-lived plasma blasts are the main B cell effector subset during the course of multiple sclerosis. Brain (2005) 128:1667–76. 10.1093/brain/awh486 15800022

[18] Cepok S von Geldern G Grummel V Hochgesand S Celik H Hartung H Accumulation of class switched IgD-IgM- memory B cells in the cerebrospinal fluid during neuroinflammation. J Neuroimmunol (2006) 180:33–9. 10.1016/j.jneuroim.2006.06.031 16952404

[19] Corcione A Casazza S Ferretti E Giunti D Zappia E Pistorio A Recapitulation of B cell differentiation in the central nervous system of patients with multiple sclerosis. Proc Natl Acad Sci USA (2004) 101:11064–9. 10.1073/pnas.0402455101 PMC503741 15263096

[20] Duddy M Niino M Adatia F Hebert S Freedman M Atkins H Distinct effector cytokine profiles of memory and naive human B cell subsets and implication in multiple sclerosis. J Immunol (2007) 178:6092–9. 10.4049/jimmunol.178.10.6092 17475834

[21] Haas J Bekeredjian-Ding I Milkova M Balint B Schwarz A Korporal M B cells undergo unique compartmentalized redistribution in multiple sclerosis. J Autoimmun (2011) 37:289–99. 10.1016/j.jaut.2011.08.003 21924866

[22] Schwerk C Papandreou T Schuhmann D Nickol L Borkowski J Steinmann U Polar invasion and translocation of neisseria meningitidis and streptococcus suis in a novel human model of the blood–cerebrospinal fluid barrier. PloS One (2012) 7:e30069. 10.1371/journal.pone.0030069 22253884 PMC3256222

[23] Schneider H Weber CE Schoeller J Steinmann U Borkowski J Ishikawa H Chemotaxis of T cells after infection of human choroid plexus papilloma cells with Echovirus 30 in an in vitro model of the blood-cerebrospinal fluid barrier. Virus Res (2012) 170:66–74. 10.1016/j.virusres.2012.08.019 23000117

[24] Steinmann U Borkowski J Wolburg H Schröppel B Findeisen P Weiss C Transmigration of polymorphnuclear neutrophils and monocytes through the human blood-cerebrospinal fluid barrier after bacterial infection in vitro. J Neuroinflam (2013) 10:31. 10.1186/1742-2094-10-31 PMC3663685 23448224

[25] Dinner S Borkowski J Stump-Guthier C Ishikawa H Tenenbaum T Schroten H A Choroid Plexus Epithelial Cell-based Model of the Human Blood-Cerebrospinal Fluid Barrier to Study Bacterial Infection from the Basolateral Side. Vis Exp (2016) 111:1–10. 10.3791/54061 PMC4942071 27213495

[26] Polman CH Reingold SC Banwell B Clanet M Cohen JA Filippi M Diagnostic criteria for multiple sclerosis: 2010 Revisions to the McDonald criteria. Ann Neurol (2011) 69:292–302. 10.1002/ana.22366 21387374 PMC3084507

[27] Ishiwata I Ishiwata I Ishiwata E Sato Y Kiguchi K Tachibana Z Establishment and characterization of a human malignant choroids plexus papilloma cell line (HIBCPP). Hum Cell (2005) 18:67–72. 10.1111/j.1749-0774.2005.tb00059.x 16130902

[28] Balint B Haas J Schwarz A Jarius S Fürwentsches A Engelhardt K T-cell homeostasis in pediatric multiple sclerosis: old cells in young patients. Neurology (2013) 81:784–92. 10.1212/WNL.0b013e3182a2ce0e 23911752

[29] Storch-Hagenlocher B Haas J Vogt-Schaden ME Bentz M Hoffmann LA Biessmann A Molecular analysis of the CDR3 encoding region of the immunoglobulin heavy chain locus in cerebrospinal fluid cells as a diagnostic tool in lymphomatous meningitis. Ann Neurol (2000) 47:211–7. 10.1002/1531-8249(200002)47:2<211::AID-ANA11>3.0.CO;2-9 10665492

[30] Michel L Touil H Pikor NB Gommerman JL Prat A Bar-Or A B Cells in the Multiple Sclerosis Central Nervous System: Trafficking and Contribution to CNS-Compartmentalized Inflammation. Front Immunol (2015) 6:636. 10.3389/fimmu.2015.00636 26732544 PMC4689808

[31] Palanichamy A Apeltsin L Kuo TC Sirota M Wang S Pitts SJ Immunoglobulin class-switched B cells form an active immune axis between CNS and periphery in multiple sclerosis. Sci Transl Med (2014) 6:248ra106. 10.1126/scitranslmed.3008930 PMC4176763 25100740

[32] Schwartz M Baruch K The resolution of neuroinflammation in neurodegeneration: leukocyte recruitment via the choroid plexus. EMBO J (2014) 33:7–22. 10.1002/embj.201386609 24357543 PMC3990679

[33] Gründler T Quednau N Stump C Orian-Rousseau V Ishikawa H Wolburg H The surface proteins InlA and InlB are interdependently required for polar basolateral invasion by Listeria monocytogenes in a human model of the blood-cerebrospinal fluid barrier. Microbes Infect (2013) 15:291–301. 10.1016/j.micinf.2012.12.005 23376167

[34] Tenenbaum T Steinmann U Friedrich C Berger J Schwerk C Schroten H Culture models to study leukocyte trafficking across the choroid plexus. Fluids Barriers CNS (2013) 10:1. 10.1186/2045-8118-10-1 23305147 PMC3560101

[35] Dahm T Adams O Boettcher S Diedrich S Morozov V Hansman G Strain-dependent effects of clinical echovirus 30 outbreak isolates at the blood-CSF barrier. J Neuroinflam (2018) 15:50. 10.1186/s12974-018-1061-4 PMC5819246 29463289

[36] Bar-Or A Fawaz L Fan B Darlington PJ Rieger A Ghorayeb C Abnormal B-cell cytokine responses a trigger of T-cell-mediated disease in MS? Ann Neurol (2010) 67:452–61. 10.1002/ana.21939 20437580

[37] Kowarik MC Pellkofer HL Cepok S Korn T Kümpfel T Buck D Differential effects of fingolimod (FTY720) on immune cells in the CSF and blood of patients with MS. Neurology (2011) 76:1214–21. 10.1212/WNL.0b013e3182143564 21464424

[38] Lohmann L Janoschka C Schulte-Mecklenbeck A Klinsing S Kirstein L Hanning U Immune Cell Profiling During Switching from Natalizumab to Fingolimod Reveals Differential Effects on Systemic Immune-Regulatory Networks and on Trafficking of Non-T Cell Populations into the Cerebrospinal Fluid-Results from the ToFingo Successor Study. Front Immunol (2018) 9:1560. 10.3389/fimmu.2018.01560 30050529 PMC6052886

[39] Lehmann-Horn K Sagan SA Bernard CC Sobel RA Zamvil SS B-cell very late antigen-4 deficiency reduces leukocyte recruitment and susceptibility to central nervous system autoimmunity. Ann Neurol (2015) 77:902–8. 10.1002/ana.24387 PMC4405474 25712734

[40] Baruch K Ron-Hareln N Gal H Deczkowska A Shifrut E Ndifon W CNS-specific immunity at the choroid plexus shifts toward destructive Th2 inflammation in brain aging. Proc Natl Acad Sci USA (2013) 110:2264–9. 10.1073/pnas.1211270110 PMC3568380 23335631

[41] Alter A Duddy M Hebert S Biernacki K Prat A Antel JP Determinants of human B cell migration across brain endothelial cells. J Immunol (2003) 170:4497–505. 10.4049/jimmunol.170.9.4497 12707326

[42] Kooij G Kopplin K Blasig R Stuiver M Koning N Goverse G Disturbed function of the blood-cerebrospinal fluid barrier aggravates neuro-inflammation. Acta Neuropathol (2014) 128:267–77. 10.1007/s00401-013-1227-1 24356983

[43] Engelhardt B Wolburg H Mini-review: Transendothelial migration of leukocytes: through the front door or around the side of the house? Eur J Immunol (2004) 34:2955–63. 10.1002/eji.200425327 15376193

[44] von Büdingen HC Kuo TC Sirota M van Belle CJ Apeltsin L Glanville J B cell exchange across the blood-brain barrier in multiple sclerosis. J Clin Invest (2012) 122:4533–43. 10.1172/JCI63842 PMC3533544 23160197

[45] Baker D Marta M Pryce G Giovannoni G Schmierer K Memory B Cells are Major Targets for Effective Immunotherapy in Relapsing Multiple Sclerosis. EBioMedicine (2017) 16:41–50. 10.1016/j.ebiom.2017.01.042 28161400 PMC5474520

