# Supplementary material for: The Choroid Plexus Is Permissive for a Preactivated Antigen-Experienced Memory B-Cell Subset in Multiple Sclerosis
Source: Front Immunol. 2021 Jan 26;11:618544. doi: 10.3389/fimmu.2020.618544 (PMC7870993; doi:10.3389/fimmu.2020.618544)
Supplement: Supplementary file 1 [file DataSheet_1.doc]

Supplementary Materials


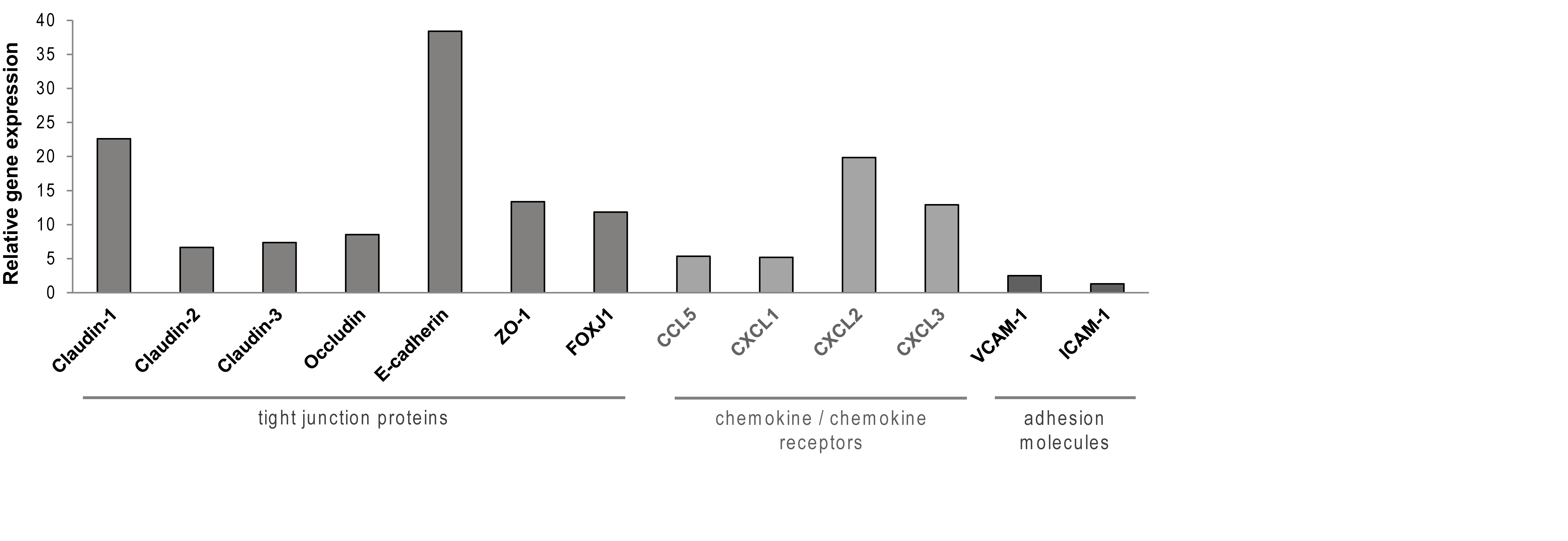


**Supplementary Figure 1.** Constitutive gene expression in HIBCPP cells. Constitutive expression of genes encoding for tight junction proteins, chemokines / chemokine receptors and adhesion molecules in fresh and unstimulated HIBCPP cells as determined by real-time PCR. mRNA expression levels were normalized to GAPDH transcripts and relative expression was determined relative to the gene with the lowest expression. Bars represent means of three independent experiments.


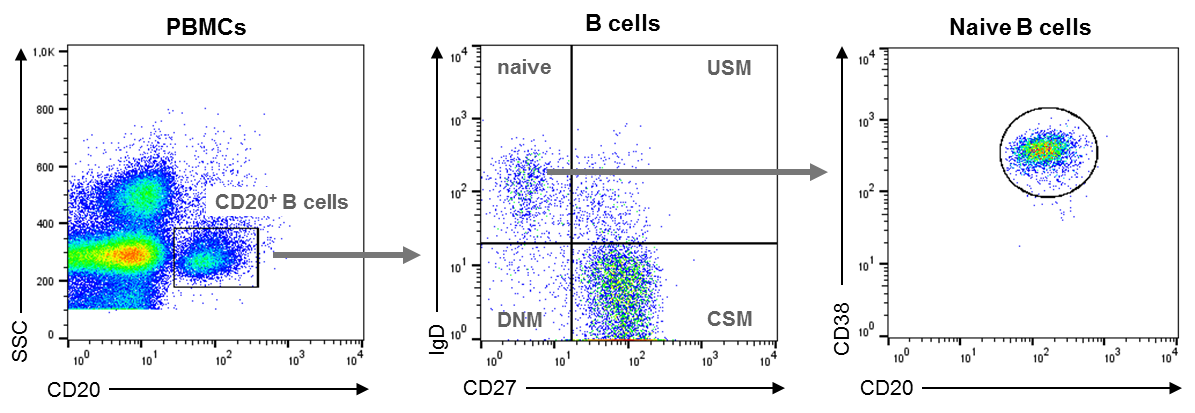


**Supplementary Figure 2.** Flow cytometric analysis of B lymphocytes in peripheral blood. Stained PBMCs were first gated for CD20^+^ (total B cells), followed by CD27/IgD dot plot analysis allowing to identify CD27^-^IgD^+^ naïve, CD20^-^CD27^-^IgD^-^ double negative memory (DNM), CD20^+^CD27^+^IgD^-^ class switched memory (CSM), CD20^+^CD27^+^IgD^+^ non-switched memory B cells (USM) and CD20^+^CD27^-^IgD^+^CD38^+^ transitional (TN) B-cell subtypes.

**
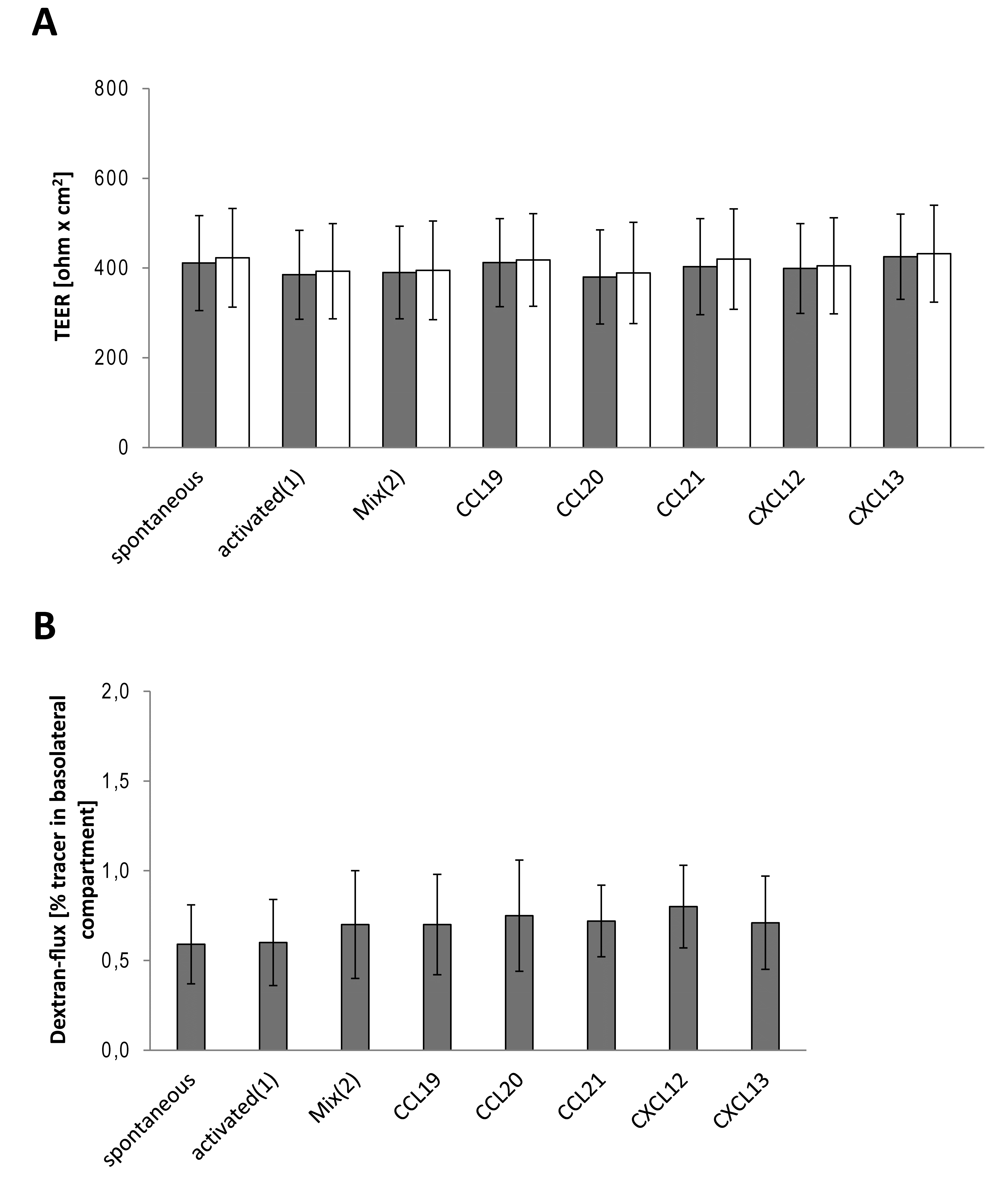
**

**Supplementary Figure 3**. Barrier characteristics of HIBCPP after transmigration of B lymphocytes. (**A**) B-cell transmigration did not alter barrier function of the HIBCPP cells as determined by measuring the TEER values before and after 4 hours of TM. (**B**) Paracellular permeability of HIBCPP cells was further assessed via measurement of the passage of Dextran-TexasRed tracer solution from the upper to the lower compartment of cell culture inserts during the course of TM experiments as previously described (33). Percentages of tracer in the lower compartment constantly stayed below 2%, thereby confirming tight HIBCPP cell barriers throughout all TM experiments. Shown is the mean ± SD of six independent experiments (each carried out in triplicates) for both TEER and Dextran. ^(1)^CD40 mAb [5 µg/ml] + IgM [12.5 µg/ml]; no chemokines; ^(2)^CD40/IgM plus CCL19 [500 ng/ml], CCL20 [100 ng/ml], CCL21 [120 ng/ml], CXCL12 [100 ng/ml], and CXCL13 [1 µg/ml]; ^(3)^CD40/IgM plus indicated chemokine.

**
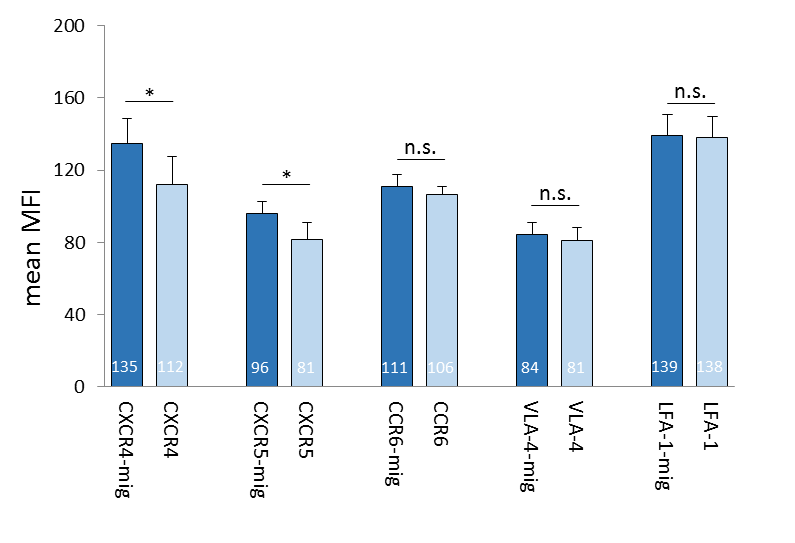
**

**Supplementary Figure 4.** Surface expression of CXCR4, CXCR5, CCR6, VLA-4 and LFA-1 on migrated and non-migrated CSM B cells obtained from healthy donors (*n* = 10) as determined by flow cytometry. Bars denote mean fluorescence intensities (MFIs) of surface stainings for CXCR4, CXCR5, CCR6, VLA-4 and LFA-1 on migrated (blue bars) and non-migrated (light blue bars) CSM B cells. Standard deviations and significances are indicated (**p* < 0.05, n.s. = non-significant; one-way ANOVA plus post Hoc Tukey test).

**
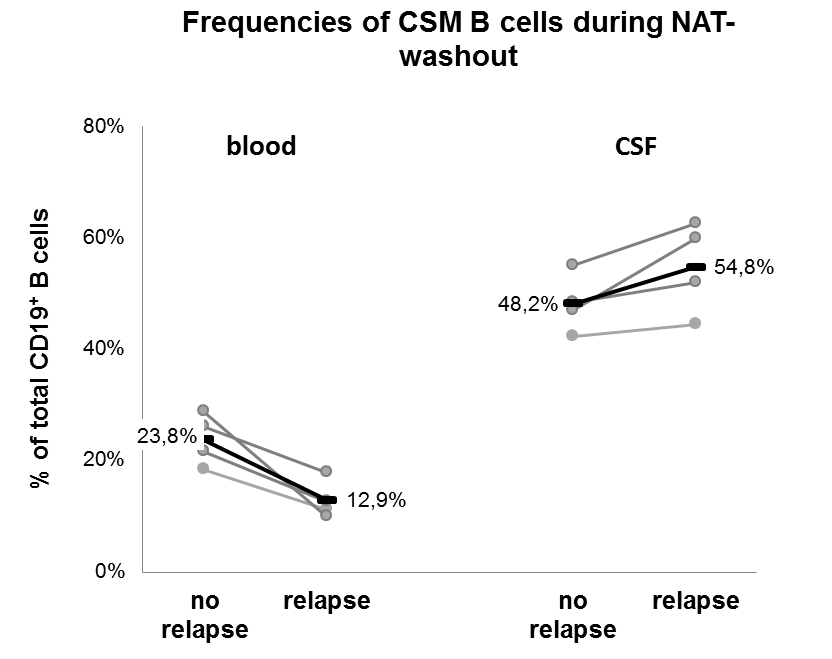
**

**Supplementary Figure 5.** Frequencies of class switched memory (CSM) B cells in peripheral blood and cerebrospinal fluid (CSF) of MS patients during washout after natalizumab (NAT) withdrawal. As determined by flow cytometric analysis relative frequencies of circulating CD20^+^CD27^+^IgD^-^ CSM markedly dropped in blood along with an increase in parallel CSF samples in patients experiencing a relapse (*n* = 4). Means are indicated.

| **Gene symbol** | **Description** | **NCBI Reference Sequence** |
| --- | --- | --- |
| CD40 | TNF receptor superfamily member 5 | NM_001250 |
| CD81 | 26 kDa cell surface protein TAPA-1 | NM_004356 |
| ICOSLG | Inducible T-cell co-stimulator ligand | NM_015259 |
| MS4A1 | CD20, membrane spanning 4-domains A1 | NM_021950 |
| CD27 | TNF receptor superfamily member 7 | NM_001242 |
| CXCR4 | Chemokine (C-X-C motif) receptor 4 | NM_003467 |
| HPRT1 | Hypoxanthin-guanin-phosphoribosyltransferase 1 | Housekeeping gene |
| RTC | Reverse transcription internal control | Control |
| CD40LG | CD40 ligand | NM_000074 |
| FAS | TNF receptor superfamily, member 6 | NM_000043 |
| IL6 | Interleukin 6 (interferon beta 2) | NM_000600 |
| TGFB1 | Transforming growth factor beta 1 | NM_000660 |
| BLNK | B-cell linker | NM_013314 |
| CXCR5 | Chemokine (C-X-C motif) receptor 5 | NM_001716 |
| RPLP0 | Ribosomal protein lateral stalk subunit P0 | Housekeeping gene |
| PPC | PCR positive control | Control |
| CD80 | B7-1 receptor | NM_005191 |
| FASLG | Fas ligand | NM_000639 |
| IL10 | Interleukin 10 | NM_000572 |
| BCL2 | B-cell CLL/lymphoma 2 | NM_000633 |
| RAG1 | Recombination activating gene 1 | NM_000448 |
| CCR6 | Chemokine (C-C motif) receptor 6 | NM_004367 |
| CXCR7 | Chemokine (C-X-C motif) receptor 5 | NM_020311 |
| GDC | Genomic DNA contamination control | Control |

**Supplementary Table 1.** Gene table of custom human RT² Profiler™ PCR array.

| **Name** | **Forward sequence** | **Reverse sequence** |
| --- | --- | --- |
| CCL5 | 5'-TACCATGAAGGTCTCCGC-3' | 5'-GACAAAGACGACTGCTGG-3' |
| Claudin-1 | 5'-GAAGATGAGGATGGCTGTCA-3' | 5'-AAATTCGTACCTGGCATTGA-3' |
| Claudin-2 | 5'-ACCATTCCTTGACGGTGTCTA-3' | 5'-GCTGATTTTCCATTACGCCT-3' |
| Claudin-3 | 5'-AACACCATTATCCGGGACTTCT-3' | 5'-CGCGGAGTAGACGACCTTG-3' |
| CXCL1 | 5'-CTCTTCCGCTCCTCTCAC-3' | 5'-GGGGACTTCACGTTCACACT-3' |
| CXCL2 | 5'-CTCAAGAATGGGCAGAAAGC-3' | 5'-AAACACATTAGGCGCAATCC-3' |
| CXCL3 | 5'-CGCCCAAACCGAAGTCATAG-3' | 5'-GCTCCCCTTGTTCAGTATCTTTT-3' |
| E-cadherin | 5'-CCTGCCAATCCCGATGA-3' | 5'-TGCCCCATTCGTTCAAGTA-3' |
| FOXJ1 | 5'-CCTCCCTACTCGTATGCCAC-3' | 5'-CGAGGCACTTTGATGAAGCAC-3' |
| GAPDH | 5'-GTTCGACAGTCAGCCGCATC-3' | 5'-GGAATTTGCCATGGGTGGA-3' |
| ICAM1 | 5'-CACAGGCCGCCACTAACAA-3' | 5'-GGTTCCATTGATCCAGGTCTT-3' |
| Occludin | 5'-AGGAACACATTTATGATGAGCAG-3' | 5'-GAAGTCATCCACAGGCGAA-3' |
| VCAM1 | 5'-GCACGAGCTTCCTGAGCACTT-3' | 5'-CTGTGTGACGAGGAAACAATG-3' |
| ZO1 | 5'-GCCAAGCAATGGCAGTCTC-3' | 5'-CTGGGCCGAAGAAATCCCATC-3' |

**Supplementary Table 2.** List of primers used in Real time-PCR analysis.

|  | **HC**  *n* = 15 | **MS**  Relapse  untreated  *n* = 10 | **MS**  Remission  untreated  *n* = 8 | **MS**  Remission FIN  *n* = 5 | **MS**  Remission NAT  *n* = 5 |
| --- | --- | --- | --- | --- | --- |
| Total B cells* | 5.2 ± 1.7 | 4.3 ± 1.6 | 5.6 ± 2.4 | 3.8 ± 2.2 | 11.3 ± 4.0 |
| Naive^†^ | 56.2 ± 14.6 | 62.4 ± 13.4 | 57.2 ± 11.4 | 69.6 ± 10.2 | 43.7 ± 10.8 |
| TN^†^ | 4.3 ± 1.5 | 5.8 ± 3.3 | 4.9 ± 2.5 | 6.6 ± 3.2 | 2.7 ± 1.8 |
| CSM^†^ | 23.1 ± 10.1 | 18.4 ± 8.7 | 20.5 ± 8.9 | 12.2 ± 5.4 | 26.2 ± 7.5 |
| USM^†^ | 15.0 ± 9.0 | 10.5 ± 6.3 | 13.0 ± 6.2 | 6.9 ± 3.2 | 19.0 ± 8.4 |
| DNM^†^ | 5.4 ± 3.9 | 7.7 ± 4.0 | 7.3 ± 3.6 | 11.6 ± 5.2 | 9.9 ± 3.8 |

**Supplementary Table 3.** Frequencies of B-cell subsets in blood of study patients and controls. FIN = Fingolimod, NAT = Natalizumab, TN = transitional, CSM = class switched memory, USM = non-switched memory DNM = double negative memory B cells. Data are given as mean ± SD. *% of PBMC, ^†^% of CD20^+^ B cells.

|  | **A) Migrated vs. non-migrated B cells**  *n* = 15 HC | | **B) Migrated vs. non-migrated B cells**  *n* = 10 MS patients | | **C) CSF-derived vs. blood-derived B cells**  *n* = 10 MS patients | |
| --- | --- | --- | --- | --- | --- | --- |
| **Gene symbol** | **x-fold** | ***P*-value** | **x-fold** | ***P*-value** | **x-fold** | ***P*-value** |
| CD40 | -2.0 | 0.049 | -4.6 | 0.007 | -3.1 | < 0.001 |
| CD81 | 2.1 | 0.018 | 2.4 | 0.057 | 2.6 | 0.045 |
| ICOSLG | 0.9 | 0.366 | 1.3 | 0.210 | -1.4 | 0.487 |
| MS4A1 | -2.9 | 0.019 | -2.5 | 0.020 | -5.5 | < 0.001 |
| CD27 | 1.6 | 0.495 | 2.2 | 0.071 | 2.5 | 0.038 |
| CXCR4 | 6.0 | 0.027 | 5.8 | 0.029 | 3.5 | 0.028 |
| CD40LG | 2.2 | 0.019 | 2.7 | 0.080 | 5.0 | 0.014 |
| FAS | 1.8 | 0.319 | 1.5 | 0.374 | 2.7 | 0.275 |
| IL6 | -1.2 | 0.669 | 1.2 | 0.336 | -1.6 | 0.678 |
| TGFB1 | 1.4 | 0.251 | -1.01 | 0.360 | -2.7 | 0.021 |
| BLNK | -2.6 | 0.311 | -1.9 | 0.559 | -1.2 | 0.819 |
| CXCR5 | 3.6 | 0.013 | 5.2 | 0.028 | 2.7 | 0.036 |
| CD80 | 1.2 | 0.118 | 1.8 | 0.088 | 2.3 | 0.039 |
| FASLG | -1.2 | 0.857 | -1.8 | 0.354 | 2.0 | 0.477 |
| IL10 | 6.5 | 0.010 | 3.3 | 0.048 | 2.2 | 0.010 |
| BCL2 | -2.4 | 0.091 | -1.1 | 0.186 | -4.1 | 0.033 |
| RAG1 | -3.4 | 0.312 | -6.0 | 0.022 | -1.7 | 0.500 |
| CCR6 | 1.9 | 0.156 | 2.8 | 0.113 | -0.2 | 0.620 |
| CXCR7 | 1.7 | 0.225 | 1.4 | 0.171 | 1.5 | 0.686 |

**Supplementary Table 4.** Results and statistics of custom human RT² Profiler™ PCR arrays. Gene expression profile of B cells obtained from peripheral blood of (A) 15 healthy donors and (B) ten MS patients with active disease after TM across HIBCPP cells as determined by PCR array. X-fold represents expression levels of genes involved in B-cell activation, trafficking and apoptosis in B cells that have migrated through the HIBCPP layer when compared to B cells that have not migrated. (C) Gene expression profile of CSF derived B cells. X-fold represents gene expression levels in total B cells isolated from CSF samples obtained from ten MS patients with active disease when compared to B cells derived from parallel blood samples. Data are presented as the means from three separate experiments.
